# AGEomics Biomarkers and Machine Learning—Realizing the Potential of Protein Glycation in Clinical Diagnostics

**DOI:** 10.3390/ijms23094584

**Published:** 2022-04-21

**Authors:** Naila Rabbani

**Affiliations:** Department of Basic Medical Science, College of Medicine, QU Health, Qatar University, Doha P.O. Box 2713, Qatar; n.rabbani@qu.edu.qa; Tel.:+974-4403-7849

**Keywords:** glycation, machine learning, AGEomics, autism, diabetes, arthritis, Alzheimer’s disease, Parkinson’s disease

## Abstract

Protein damage by glycation, oxidation and nitration is a continuous process in the physiological system caused by reactive metabolites associated with dicarbonyl stress, oxidative stress and nitrative stress, respectively. The term AGEomics is defined as multiplexed quantitation of spontaneous modification of proteins damage and other usually low-level modifications associated with a change of structure and function—for example, citrullination and transglutamination. The method of quantitation is stable isotopic dilution analysis liquid chromatography—tandem mass spectrometry (LC-MS/MS). This provides robust quantitation of normal and damaged or modified amino acids concurrently. AGEomics biomarkers have been used in diagnostic algorithms using machine learning methods. In this review, I describe the utility of AGEomics biomarkers and provide evidence why these are close to the phenotype of a condition or disease compared to other metabolites and metabolomic approaches and how to train and test algorithms for clinical diagnostic and screening applications with high accuracy, sensitivity and specificity using machine learning approaches.

## 1. Introduction

**Figure 1:**
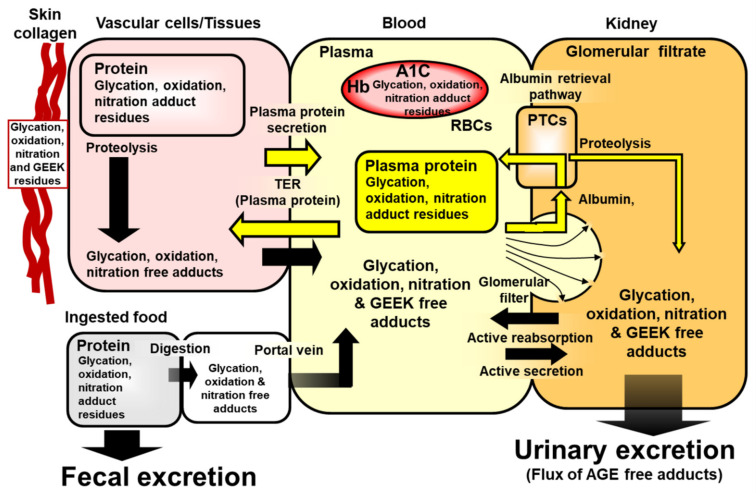
Schematic multicompartment representation of the formation, physiological processing and transit of protein glycation, oxidation, nitration and GEEK adducts in mammalian metabolism. Abbreviations: A1C, glycated hemoglobin HbA1c; PTC, proximal tubular epithelial cell; TER, transcapillary escape rate. Modified from a similar scheme for glycation adducts in [5]. Adapted with permission from Ref. [5]. Copyright year 2021, Elsevier.

It is commonly considered that, in the application of omics technology to the study of physiological processes, information embedded in the analytical data is increasingly closer to the disease phenotype in progressing from analysis based on genetics to transcriptomics, proteomics and metabolomics platforms [1]. For assessment of biomarkers close to the disease phenotype, therefore, it may be advantageous to analyze metabolites. However, a disadvantage is that metabolites are often short-lived, and estimates are made by analysis of samples collected at a particular time point. Therefore, we gain information on a very short time interval or snapshot of the disease. Thus, it may be beneficial to use protein-based biomarkers where major proteins in the clinical setting such as serum albumin, hemoglobin and skin collagen have approximate half-lives of ca. 20 days, 42 days and 15 years, respectively [2,3,4]. It is particularly advantageous where variation in levels of metabolites is captured over the lifespan of the protein by reaction of a metabolite with a protein to form a stable adduct. The most well-known clinical example of this is glycated hemoglobin HbA1c (A1C)—an adduct formed by the spontaneous, non-enzymatic reaction of glucose with hemoglobin. The level of HbA1c provides a report of glycemic control over the previous 90–120 days from sampling in patients with diabetes and is also used in the diagnosis of diabetes and prediabetes, reviewed in [5]. In physiological systems, proteins undergo spontaneous modifications: modification by other glycating agents to form advanced glycation endproducts (AGEs), reaction with reactive oxygen species (ROS) to form protein oxidation adducts and reaction with reactive nitrogen species to form protein nitration adducts. Change in the rate of formation and/or removal of protein glycation, oxidation and nitration adducts occurs in many chronic diseases [5,6]. There are also usually low-level enzymatic modifications of proteins such as the conversion of arginine residues to citrulline residues by peptidylarginine deiminases (PADs) [7], formation of Nε(γ-glutamyl)lysine (GEEK) crosslink by transglutaminases [8] and formation of o,o′-dityrosine crosslinks catalyzed by NADPH-dependent dual oxidase (DUOX) [9]—also formed non-enzymatically by ROS. These modifications of proteins provide a fingerprint of patterns of damage or structural and functional change at different tissue sites and in body fluid compartments (Figure 1). Optimum combination and weighting of selected glycation, oxidation and nitration adduct residues in plasma protein may provide classifier information for some of the most challenging clinical conditions to diagnose. For example, the recent application of specific and selective plasma protein glycation and oxidation adducts for a blood test to diagnose autism spectrum disorder (ASD) [10].

The proteolysis products of modified proteins, glycated, oxidized and nitrated amino acids—also called protein glycation, oxidation and nitration free adducts—are released into plasma and excreted in the urine. Unlike unmodified amino acids, they are not reincorporated into proteins. The levels of these metabolites are therefore very sensitive to changes in rates of protein modification and hydrolysis of modified proteins. An example of this is the release of protein glycation, oxidation and nitration free adducts from cartilage by increased proteolysis in early-stage arthritic disease which provided amino acid analytes for early-stage diagnosis and classification of arthritis [11,12]. The plasma concentrations of protein glycation, oxidation and nitration free adducts are also highly sensitive to renal function through high renal clearance of these analytes [13,14]. Indeed, increased fractional clearance of glycation free adducts was a powerful risk predictor of the progression of diabetic kidney disease [15]. This may reflect a decline in reuptake of glycation free adducts in the renal proximal tubules in the early stages of impairment of renal function.

The urinary excretion of protein glycation, oxidation and nitration free adducts provides an estimate of the whole-body flux of formation of these analytes, with contributions from the absorption of glycated, oxidized and nitrated amino acids from the digestion of glycated, oxidized and nitrated proteins in food [16]. Pyrraline is an AGE originating only in food [17]. Where this is measured, a correction may be applied to deduce the proportion of the flux of other protein glycation free adducts, and also the flux of oxidation and nitration free adducts produced endogenously [18]. 

In this article, I review how metabolomics and proteomics focused on the study of protein glycation, oxidation, nitration and other low-level functionally-impairing modifications of proteins, “AGEomics technology”, has an important role in the development of clinical screening diagnostics and therapeutic monitoring—particularly with the application of machine learning for the development of diagnostic algorithms.

## 2. Focused Proteomics and Metabolomics of Protein Damage—“AGEomics” and Its Utility

Biomarker selection is likely to be most beneficial when focused on mechanisms of health decline or early-stage disease process—where loss or change of protein function may be involved. Measurement of protein glycation, oxidation and nitration markers, and protein citrullination and transglutamination adduct GEEK, is applicable to early-stage disease diagnosis, progression and therapeutic monitoring. Modified amino acids are robustly quantified in a multiplexed assay using stable isotopic dilution analysis tandem mass spectrometry (LC-MS/MS), initially described in [19], and with later refinements [20,21]. Analysis of samples by stable isotopic dilution analysis LC MS/MS has the advantage that absolute quantitation of analytes is made, facilitating robust comparison between laboratories. LC-MS/MS is a widely applicable, high dynamic range multiplexed technique without carry-over between analytes. Glycation, oxidation, nitration, citrulline and GEEK adducts in proteins are assayed with prior automated enzymatic hydrolysis. Analyte content is normalized to the unmodified amino acid precursor. This provides for robust quantitation of normal and modified amino acids concurrently [19,20]. It is also applicable to the measurement of nucleotide glycation and oxidation adducts, such as 8-hydroxydeoxyguanosine [22]. Modified amino acid adduct residues may be analyzed in plasma protein and related free adducts analyzed in plasma ultrafiltrate, reported as the plasma concentration, and in urine normalized to creatinine and reported as a flux of urinary excretion. The content of adducts in plasma protein is the steady-state level influenced by the rate of protein modification and rate of protein turnover. For serum albumin, most of the protein modification occurs in the vascular compartment. It is influenced by albumin turnover–particularly in cirrhosis, transcapillary escape rate and albumin glomerular leakage and retrieval–as recently reviewed [5]. Different analytes report on different metabolic and pathogenic processes, as shown in Table 1. Note that each adduct may feature in diagnostic algorithms for multiple health conditions and diseases indicating that the related protein modification process and related metabolic status reported contributes to multiple different health conditions and diseases. The combination and weighting of the adducts together with other features provide sensitivity and specificity for the particular health status under investigation.

### 2.1. Protein Glycation

Protein glycation is the non-enzymatic modification of proteins by reaction with simple reducing sugars and related compounds. It involves early-stage glycation by glucose reacting with N-terminal and lysyl side chain amino groups to form fructosamine adducts. For lysine residues, this forms Nε(1-deoxyfructosyl)lysine (FL). Other common fructosamines are formed on the N-terminal valine residue of the ß-chain of hemoglobin in A1C and on the N-terminal aspartate residue of albumin in glycated albumin [5]. Early-stage glycation by glucose of endogenous proteins often reflects glycemic control. For FL free adducts in plasma and urine, there may be a large and varying contribution from the absorbance of FL free adducts from digested glycated proteins in food.

Other and later stages of glycation form a diverse range of adducts on N-terminal amino groups, lysine and arginine residue side chains called advanced glycation endproducts (AGEs) [43]. Nε-Carboxymethyl lysine (CML) was one of the first discovered AGEs [44] and is a major lysine-derived AGE with often high content in processed foods [45]. It is formed from the oxidative degradation of FL and other sources. The ratio of CML to FL residue content has been suggested as a marker of oxidative damage [29]. Hydroimidazolone (MG-H1) is a major arginine-derived AGE formed from methylglyoxal (MG). MG-H1 is linked to increased glycolysis, decreased activity of glyoxalase 1 (Glo1)—the major enzyme metabolizing MG [46]—and fasting and postprandial glucose exposure through MG being a by-product of glycolysis. MG-H1 free adduct is also absorbed after digestion of food proteins. The formation of MG-H1 is associated with a high risk of functional impairment of proteins and protein unfolding [18,19,47]. Glucosepane (GSP) is a major quantitative crosslink formed in protein glycation. GSP is formed from FL precursor and hence provides a stable, cumulative marker of glucose exposure [31,48,49]. GSP was increased in early stages and progressively increased further in development towards advanced stages of osteoarthritis (OA), suggesting a good biomarker for early diagnosis of OA [50]. GSP is likely not absorbed from food; that is, the urinary flux of GSP did not correlate with pyrraline [16]—see below. Pentosidine is a minor protein crosslink with intense fluorescence [51]. This is formed from pentose precursors and reflects pentosephosphate pathway metabolic activity [33]. Pyrraline is a glucose-derived AGE formed at high temperatures of culinary processing, originating only from food [17]. Free adduct is an indicator of food consumption and/or intestinal permeability [17,18]. For protein glycation, oxidation and nitration free adducts with a major contribution from food, the urinary flux of the free adduct correlates positively with urinary pyrraline. The non-zero intercept in linear regression of urinary free adduct on pyrraline gives an estimate of endogenous protein glycation, oxidation or nitration adduct formation. For example, the mean endogenous formation of MG-H1 was 13.3 nmol/mg creatinine in overweight and obese subjects. Endogenous MG-H1 formation accounted for 68% of total MG-H1 exposure, and food accounted for 32% MG-H1 exposure. However, the contribution of MG-H1 from food was highly variable [18].

### 2.2. Protein Oxidation

Methionine sulfoxide (MetSO) is an oxidative modification found widespread in physiological systems. It is formed by the oxidation of Met and Met residues of proteins by ROS, RNS and hypochlorite as a mixture of *S*- and *R*-diastereomers. MetSO Protein residue and free adduct forms are reduced to Met by methionine sulfoxide reductases with the exception of the *R*-diastereomer of MetSO free adduct [35]. α-Aminoadipic semialdehyde (AASA) is a “protein carbonyl” formed by the oxidative deamination of lysine [36,52]. The related protein carbonyl, glutamic semialdehyde (GSA), is formed by the oxidative deguanidylation of arginine and oxidative ring-opening of proline [36,52]. Dityrosine is an oxidative crosslink formed spontaneously in oxidative stress and enzymatically by DUOX [9,23]. N-Formylkynurenine (NFK) is formed non-enzymatically by the oxidation of tryptophan by hydrogen peroxide, peroxynitrite and hypochlorite [37] and enzymatically by indoleamine 2,3-dioxygenase (IDO). Increased IDO activity is involved in immunoregulation, inflammation and host defense against infectious disease [38]. These, mostly, irreversible adducts of protein oxidation are assayed in AGEomics and provide a multi-faceted fingerprint of protein oxidative damage.

### 2.3. Protein Nitration

3-Nitrotyrosine (3-NT) is the major product of protein nitration by peroxynitrite and nitryl chloride. It may report on both oxidative damage and nitric oxide availability [27,53]. Free 3-NT is metabolized in rats and human subjects, leading to the formation of the major urinary metabolite 3-nitro-4-hydroxyphenylacetic acid (3-HNA)—with the minor formation of 3-nitro-4-hydroxyphenyllactic acid and 3-nitro-4-hydroxyphenyllactic acid [54,55]. 3-NHA is not a good marker of the flux of formation of 3-NT; however, it may be formed by 3-NT independent pathways [56]. 3-NT is present in proteins of tissues, extracellular matrix, plasma and other body fluids. 3-NT residues are also present in proteins of ingested food and are absorbed as 3-NT free adducts [40].

### 2.4. Other Common Modifications

Citrulline residues are formed from selected arginine residues in proteins by peptidylarginine deiminases (PADs) [41]. Protein citrullination is linked to protein misfolding [57] and related auto-immunity, likely producing anti-cyclic citrullinated peptide (CCP) antibody positivity in early-stage rheumatoid arthritis (RA) [58]. Citrullinated protein (CP) was also increased in early-stage osteoarthritis (OA) but without anti-CCP antibody positivity [11]. 

Nε(γ-Glutamyl)lysine (GEEK) residues are formed from glutamine and lysine residues by transglutaminases (TGs). The formation of GEEK generates crosslinked supramolecular protein assemblies, particularly in the extracellular matrix. GEEK formation is involved in blood clotting, age-related impairment of elastic properties in human skin and bone formation [42]. The formation of GEEK was increased in fibroblast senescence [59]. 

## 3. Machine Learning in Protein Damage Biomarker Related Diagnostic Applications

Machine learning (ML) is a rapidly developing field where computer scientists are continually innovating and refining methods to develop algorithms. ML is intensively used in every walk of life from image analysis, voice recognition, economics, security to social media to provide customized feed and advertisements based on personal preferences. This became possible because of access to good quality data. There are also applications of ML in health care. For example, using electronic health records to generate health scores, predicting onset of disease and hospitalization for precision medicine and diagnostic algorithms [60]. In AGEomics, algorithms are developed to classify subjects–such as employed in clinical diagnostic applications for classifying case and control subjects.

In clinical diagnostics, often a single biomarker—such as A1C—does not provide conclusive classification between cases and controls. We rather need an optimum combination of biomarkers, including protein glycation, oxidation and other biomarkers, in a classifier algorithm to achieve this [10,11,12,16]. Algorithms are trained on “test set” data obtained from the initial study cohort and then the outcome is tested with data from the analysis of samples from an independent “test set” cohort. For algorithm training and testing, there are also internal validation methods that are typically used, such as five-fold cross-validation and leave-one-out analysis. Training set and test set cohort size need to be designed for adequate statistical power. A guide on power analysis in diagnostic algorithm development was published by Xia et al. [61]. The performance of the classification by the algorithm can be assessed by accuracy—ability to correctly classify cases and controls, and other conventional indicators of classification performance: sensitivity, specificity, positive likelihood ratio LR+, negative likelihood ratio LR- and others. By convention, the interpretation of LRs in terms of the level of evidence is as follows: LR+ (presence of a disease or condition): 1–2, minimal; 2–5, small; 5–10, moderate; >10, large and conclusive. LR− (absence of a disease or condition): 0.5–1.0, minimal; 0.2–0.5, small; 0.1–0.2, moderate; <0.1, large and conclusive [62]. We therefore aim for LR+ >10 and LR− <0.1 in the development of classifier algorithms. In Table 2, I give examples of where we have applied machine learning algorithm development in AGEomics: diagnosis of autism, early-stage osteoarthritis (including identification of clinical type of arthritis), a health screen for early-stage decline in metabolic, vascular and renal health, as reviewed recently [21], and a recent development for risk prediction of diabetic kidney disease [63], as shown in Table 2. Further applications are in progress. We have used different methods of algorithm development and, in general, a good approach is to try multiple algorithm development methods to identify which is best performing for the particular data set. Some algorithm development methods have optimum feature selection embedded in the training; for other algorithm development methods, filters and other assessments of the contribution of individual features to the classification performance have to be computed [64]. A method we have used, for example, is omitting features from algorithms training one-by-one and retaining features when they improve accuracy. This can also indicate the relative contribution of each feature to the overall classification accuracy [11,12]. It is best to try multiple different methods of algorithm development to identify the method most suited for the data set classification at hand.

## 4. Examples of Application of Machine Learning in Protein Damage Biomarker Related Diagnostic Using the AGEomics Platform

The first application of machine learning in diagnostic algorithm development incorporating a feature of protein modification from my group was protein citrullination in early-stage arthritis. Antibodies to CP, assessed clinically as anti-CCP antibody status was a biomarker of early-stage rheumatoid arthritis (eRA). It was assumed that protein citrullination occurred mainly in eRA [58]. Our investigation showed that CP was also prevalent in early-stage osteoarthritis (eOA) and autoimmunity to CP was a characteristic of eRA [11]. A further type of inflammatory arthritis which is normally self-resolving within a few months was termed non-RA. Combining plasma CP, the bone resorption biomarker, hydroxyproline (hyp) and anti-CCP antibody status in a 4-group classifier algorithm (classifying good skeletal health, eOA, eRA and non-RA) provided for diagnosis of both presence and type of early-stage arthritis, giving moderate evidence for the presence of eOA and eRA [11]. A better approach was to combine plasma protein glycation, oxidation and nitration-free adducts with hyp and anti-CCP antibody status. For initial classification of early-stage arthritis, any type, versus good skeletal health, algorithms were developed with LR+ = 8.3 and LR− = 0.11. 

This indicated there was moderate evidence of the presence and strong evidence of the absence of impaired skeletal health. Plasma hyp was an algorithm feature for this initial screening step. In the second step for classification of arthritis, there was strong, often conclusive evidence for the presence or absence of eOA and moderate evidence for the presence or absence of eRA and non-RA [12]. Later studies in a guinea pig model of OA revealed the glycation crosslink, GSP, was an early-stage indicator of eOA progression [50]. Glycation, oxidation and nitration free adducts are likely good diagnostic markers for early-stage arthritis as they are produced by and report on joint proteolysis [65]. They are sensitive, mechanistic biomarkers of pathogenesis because they have accumulated in joint proteins during the lifespan and, unlike unmodified amino acids, they are not reincorporated into proteins after formation. The life-long risk of developing OA of the knee is ca. 45% and the rate of progression to severe debilitating disease is ca. 4% per year [66]. There is currently no simple clinical chemistry test for diagnosis of eOA nor for assessing the risk of progression. The diagnostic algorithms developed in our studies and plasma GSP free adduct may meet this unmet clinical need [12,50]. Validation of the predictive eOA diagnostic algorithm is in progress

A further currently intractable clinical diagnostic problem and unmet need is a simple blood test for autism spectrum disorder (ASD). Autism is a developmental disorder of childhood thought to affect over 12 million people worldwide [67], with relatively high prevalence in the USA (2.47%) [68] and Europe (1.15%) [69]. Assessment for diagnosis is limited by referrals to experts in childhood development, basing diagnosis on lengthy behavioral observations and tests. Long delays of up to four years for referral of children with suspected autism are common. Early diagnosis of autism facilitates intervention with counseling and cognitive restructuring which can produce remission from symptoms [70]. In a study of 69 children with and without autism, we found higher plasma protein content of CML, CMA and DT and lower 3DG-H adduct residues in plasma protein of children with autism, compared to children with normal development. A diagnostic algorithm combining these analytes gave a test with an accuracy of 88%, sensitivity 0.92, specificity 0.84, LR+ 5.8 and LR− 0.095, indicating moderate evidence for the presence of ASD and strong, often conclusive evidence for the absence of ASD. It is therefore applicable as a screening test–particularly for subjects whose suspected ASD is false. This test is currently undergoing validation for algorithms based on amino acids and protein glycation and oxidation free adducts. The sample preparation, analysis and interpretation can be performed within a day. A simple blood test may meet the currently unmet need for improved availability of autism diagnosis. Universal screening for autism has been recommended by the American Academy of Pediatrics of children between 18 and 24 months but this has limited compliance to date. A screening test—such as the emerging blood test based on AGEomics—to select subjects for further surveillance and expert examination would help address the current unmet need for autism diagnosis [71].

In a recent application, we have shown how renal handling of glycated amino acids may produce valuable risk prediction of diabetic kidney disease. Patients with diabetes, type 1 diabetes (T1D) and type 2 diabetes (T2D), are at risk of developing diabetic kidney disease. Typically, after 10 years or less of diabetes, the initial stages of diabetic kidney disease develop—often indicated by a low-level increase in urinary albumin or microalbuminuria [72]. At this stage, treatment is initiated of all patients with diabetes with angiotensin II receptor inhibitors or blockers (ARBs) or angiotensin-converting enzyme (ACE) inhibitors to slow the rate of decline in renal function. In patients who go on to develop rapid loss of renal function–also called “early decline in renal function,” renal function declines in the range of 3–20 mL/min/year such that after 5–20 years, patients require expensive renal dialysis and have a median survival thereafter of only three years. During the decline of renal function, there is also a progressive increase in the risk of fatal cardiovascular disease, 3 to 20-fold higher than the healthy population. 

Renal function is assessed by the measurement of glomerular filtrate rate (GFR). This is the gold standard method of assessing current renal function at one or more time points during the course of diabetic kidney disease. GFR does not predict a future decline in renal function. By the time decrease in renal function is detected by GFR, diabetic kidney disease is already well-advanced. Treatment of diabetic kidney disease would be most effective if it could be given to patients when they have normal GFR, such as when microalbuminuria initially develops, and to those patients who are predicted to go on to develop an early decline in renal function. It is estimated that 19% of patients with T1D and 28% of patients with T2D develop an early decline in renal function, and this is the primary cause of progression to renal failure and the requirement for dialysis in patients with diabetes [73]. Currently, patients at risk of future renal function decline cannot be identified. Thus, in well-found healthcare systems, all patients with T1D or T2D are treated with ARBs and ACE inhibitors when they develop microalbuminuria. If patients at risk of future diabetic kidney disease could be identified, treatment could be intensified for those patients that need it to optimize slowing the rate of decline in kidney function and thereby likely avoid the need for renal dialysis and also decrease mortality from associated cardiovascular disease. Savings and avoidance of adverse effects of drug treatment could be made patients not at risk of future early decline in renal function. We measured protein glycation, oxidation and nitration free adducts in plasma and urine of 75 patients with diabetes at the point of development of microalbuminuria in the First Joslin Kidney Study [74]. These patients had 12–15 years of follow-up to assess whether they had a subsequent stable or declining renal function. We deduced the fractional excretion (FE) of damaged amino acids. FE is the rate of clearance of damaged amino acids from the blood into urine for excretion, relative to that of creatinine. We found six damaged amino acids had increased FE in patients with subsequent early decline in renal function. This reflects early-stage impairment of renal tubular re-uptake of damaged amino acids when GFR is normal. These processes later increase and contribute to future early decline in renal function. None of the plasma, urine or deduced FE values for damaged amino acids were able to discriminate conclusively between patients with future stable renal function (non-decliners) and patients with future early decline in renal function (decliners) [15]. Using machine learning to deduce algorithms with the optimum combination of damaged amino acid analytes and conventional clinical measurements, we could classify non-decliners and decliners with LR+ = 11.0 with features: A1C, log(urinary albumin-creatinine ratio), FE_Nω-carboxymethylarginine_ and FE_Glyoxal-derived hydroimidazolone_ and the plasma concentration of CML–Table 2. With the measurement of three glycated amino acids in plasma and urine, therefore, we were able to classify patients who later developed diabetic kidney disease [63]. The diagnostic power is likely based on reporting of early decline in the functional activity of amino acid cation transporters in the renal proximal tubules, with some linked to the rapid decline of GFR in genome-wide association studies [75,76]. This requires further validation and application to patients with T2D and non-diabetic kidney disease. It may offer a relatively simple test for risk prediction of diabetic kidney disease.

Alternative approaches in assessing links of protein damage markers to health conditions or disease have been to compute single or composite z-scores of glycation adduct residues in plasma protein or free adducts in serum or plasma. AGE content data is often log-transformed and, particularly in studies of diabetes and vascular complications of diabetes, adjusted for level of A1C at baseline and follow-up sample collection. In prospective studies, a related hazard ratio-an estimate of the occurrence of health conditions in the cases versus control study groups may then be deduced. This approach was used to study the association of plasma protein AGEs, CML, CEL and pentosidine residues, with cardiovascular disease in patients with T2D [77] A higher AGE score was associated with low renal function (estimated glomerular filtration rate), low BMI and low risk of peripheral artery disease. In a further study, Genuth et al. measured furosine—a surrogate analyte of FL—and six AGE residues, fluorescence, acid solubility and pepsin digestibility of skin collagen. Sample collection was made at the closeout of the Diabetes Control and Complications Trial (DCCT) study. This ten-marker panel was explored in producing an improved statistical model for risk prediction of progression microvascular complications of diabetes—diabetic nephropathy, diabetic retinopathy and diabetic neuropathy. This improved risk prediction of progression risk of retinopathy and neuropathy but not nephropathy; GSP and MG-H1 were major risk predictors. Increased furosine was linked to the worsening of all microvascular complications. Possible mechanisms underlying these associations were discussed previously [78]. Recently, CML, CEL, G-H1, MG-H1 and 3DG-H free adducts were measured in plasma and serum from the Action to Control Cardiovascular Risk in Diabetes (ACCORD) (*n* = 1,150) and Veterans Affairs Diabetes Trial (VADT) (*n* = 447). A composite AGE z score was computed and related to risk prediction of a decline in renal function [79]. A previous study on Native Americans with T2D found an association of decline in renal function with MG-H1 and CEL free adducts [80]. The advantage of the machine learning approach to classifying cases and controls is the simplicity of data inputs and ease of interpretation of diagnostic outputs.

## 5. Future Perspectives

The application of machine learning in AGEomics is providing classifier algorithms with high confidence outcomes for screening, diagnosis and risk prediction of disease or conditions. Establishing the LC-MS/MS platform for quantitation of damaging modifications is now showing potential utility in addressing some challenging problems in clinical diagnostics. There remain, however, barriers to widespread use in the clinical setting.

LC-MS/MS is a common analytical platform in the research laboratory but is yet to become routinely used in clinical chemistry laboratories. The barrier to this is limited automation, limited commercial availability of analytical standards and limited regulatory approval of LC-MS/MS-based clinical diagnostics methods. With LC-MS/MS based AGEomics now providing solutions to some intractable and high clinical diagnostics problems, such as a blood test for autism and early-stage detection osteoarthritis and classification of arthritis. There are now requirements and advantages to broadening access and use of LC-MS/MS by improving automation of instrumentation and availability of analytical standards. With interest and investments from the biotechnology and commercial sectors, increasing regulatory approval of LC-MS/MS-based clinical diagnostics will likely follow.

For clinical take-up of diagnostic methods, it is important that diagnostic algorithms are validated in both research and clinical settings. For example, assessment of the accuracy of diagnostic algorithms with clinical samples of blinded class and at the case-to-control ratio found in clinical practice. There needs to be full disclosure of the data used in algorithm training and testing and flexibility for further algorithm refinement through experience with new clinical data–which may be specific for the population studied. Currently, there is little refinement of algorithms used in clinical diagnostics after regulatory approval [81]. We are currently in a period of rapidly expanding application of machine learning in clinical diagnostics and AGEomics has a unique and important contribution to make.

For disease applications, coronary heart disease (CHD), linked to increased risk of atherosclerosis may be amenable to an AGEomics approach. The risk of CHD is linked to increased small dense LDL [82] and decreased HDL [83]. MG modification of apolipoprotein in LDL led to the formation of small dense LDL [84] and MG modification of apolipoprotein-A1 of HDL destabilized the HDL particle leading to a decreased half-life of HDL [85]. If LC-MS/MS detection of tryptic peptides or immunoassays of functionally important MG-modification sites may be developed for MG-modified LDL and HDL, these may be risk predictors of CHD. 

A further application of AGEomics is neurological disorders, such as Alzheimer’s disease (AD) and Parkinson’s disease. These are neurological disorders of major social impact. AD is the most common neurodegenerative disease and most common form of dementia worldwide [86]. Parkinson’s disease (PD) is the second most common neurological disorder after AD and is growing faster than AD. It is characterized by α-synuclein aggregation and the loss of dopaminergic neurons, resulting in a combination of motor and non-motor symptoms [87,88]. We studied protein glycation, oxidation and nitration markers of in-life cerebrospinal fluid samples and found increased MG-H1 and 3-NT free adducts in subjects with AD [89]. Increased AGEs in AD were proposed to be causative for ß-amyloid formation and cytotoxicity. For example, MG-modified ß-amyloid had enhanced neuronal toxicity [90]. Deposition of ß-amyloid correlates with increased glycolysis [91]. This might be reflected in increased formation of MG, MG-modified protein and, after proteolysis, MG-H1 free adduct in cerebrospinal fluid and release into plasma and excretion in urine. In the resting state, glucose metabolism by the brain is a major component of whole-body glucose metabolism [92]. Therefore, urinary MG-H1 collected in the morning after voiding before overnight sleeping, and corrected for the contribution of MG-H1 from food [18], may be an indicator of increased CNS glycolysis. This could be explored as a urinary screen for the detection of early-stage AD. In contrast, early-stage Parkinson’s disease is considered to be associated with decreased glucose metabolism [93,94] and may, under similar sample collection conditions, give decreased endogenous MG-H1 free adduct formation. This could be explored as a urinary screen for the detection of early-stage Parkinson’s disease.

AGEomics offers a deeper insight into dysfunctional metabolism and proteostasis than conventional omics technologies in that it reflects a covalent interaction between metabolomics and proteomics. Through this, it is able to provide a report on metabolic dysfunction over time, rather than a snapshot with conventional ‘omics technologies. This is widely exploited currently with the use of A1C for diagnosis of prediabetes and diabetes and glycemic control in diabetes. As indicated above, going beyond A1C, much wider and also clinically valuable diagnostic contributions are available.

## Figures and Tables

**Table 1:** Protein glycation, oxidation, nitration and other modification adducts assayed in AGEomics.

Modification Process	Modified Amino Acid	Reporting Characteristic	Example of Analysis and Levels ^1^
Early-stage glycation (formation of fructosamine adducts) ^2^	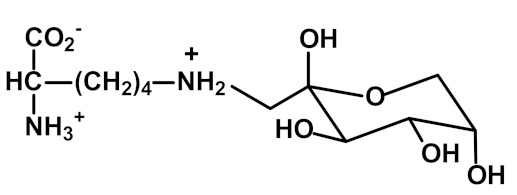 N_ε_-Fructosyl-lysine (FL)	Early-stage glycation adduct formed from glucose, reporting on exposure to increased glucose concentration [23]. Repaired intracellularly by fructosamine 3-phosphokinase [24]. FL free adduct is absorbed after digestion of food proteins [25].	Hb, 0.84 ± 0.30 mmol/mol lys; and Plasma protein, 1.35 ± 0.16 nmol/mol lys [26]. Used as markers of glycemic control in Hb (with N-terminal valine adducts) and albumin (with N-terminal aspartate adducts [5]. Urinary excretion: 26.5 (17.3–39.4) nmol/mg creatinine [16]
Advanced-stage glycation (formation of AGEs)	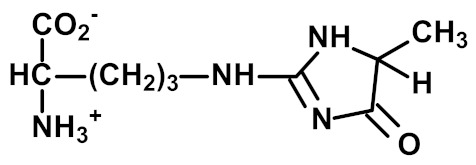 Methylglyoxal-derived hydroimidazolone (MG-H1)	A major quantitative arginine-derived AGE formed from methylglyoxal. Linked to increased fasting and postprandial glucose exposure, insulin resistance and cardiovascular disease [18,26,27,28]. MG-H1 free adduct is absorbed after digestion of food proteins [18].	Hb, 2.62 ± 0.60 mmol/mol arg; and Plasma protein, 0.31 ± 0.20 nmol/mol arg [26]. Urinary excretion: 20.1 (16.3–30.6) nmol/mg creatinine; endogenous formation 13.4 ± 2.1 nmol/mg creatinine [18]
	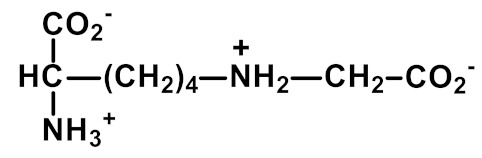 N_ε_-Carboxymethyl-lysine (CML)	Major lysine-derived AGE. Formed by the oxidative degradation of FL and other sources. CML/FL ratio is an indicator of oxidative stress [29]. CML free adduct is absorbed after digestion of food proteins [30].	Hb, 0.075 ± 0.023 mmol/mol lys; and Plasma protein, 0.038 ± 0.010 mmol/mol lys [26].
	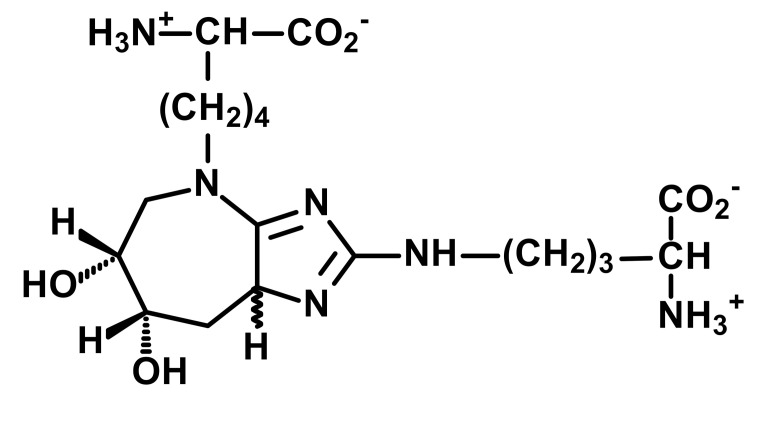 Glucosepane	Major quantitative crosslink formed in protein glycation by the degradation of FL residues [31].	Urinary excretion: 2.84 (2.41–3.36) nmol/mg creatinine [16]. Plasma free adduct increased in early-stage osteoarthritis [32].
	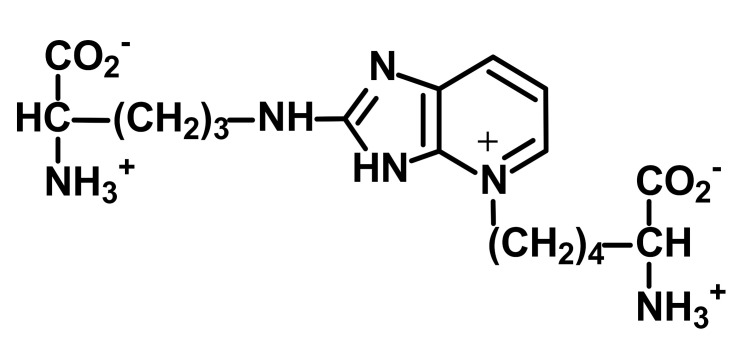	Low-level pentose sugar-derived glycation crosslink and intense fluorophore. Considered to reflect pentosephosphate pathway activity [33].	Urinary excretion: 0.258 (0.207–0.287) nmol/mg creatinine [16]. Urinary excretion is risk predictor of diabetic kidney disease [15].
	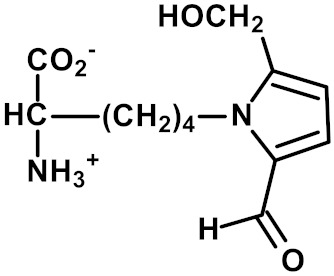 Pyrraline	Glucose-derived AGE formed at high temperatures of culinary processing; originating only from food [17,34].	Urinary excretion: 9.11 (5.69–13.67) nmol/mg creatinine in second void urine after overnight fasting [16].
Oxidation	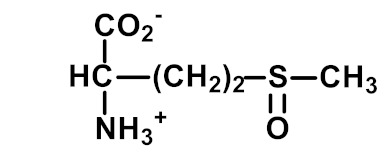 Methionine sulfoxide (MetSO; methionine-*S*-sulfoxide and methionine-*R*-sulfoxide)	Formed by the oxidation of Met and Met residues of proteins by ROS and RNS as a mixture of *S*- and *R*- diastereomers. Protein and free adduct forms are reduced to Met by methionine sulfoxide reductases, with the exception of the *R*-MetSO free adduct [35].	Hb, 2.97 ± 0.55 mmol/mol met; and Plasma protein, 0.98 ± 0.13 nmol/mol met [26].
	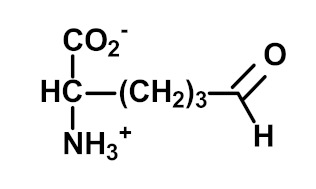 α-Aminoadipic semialdehyde (AASA)	“Protein carbonyl” formed by the oxidative deamination of lysine [36]	Plasma protein: 0.15 ± 0.05 mmol/mol lys [10]
	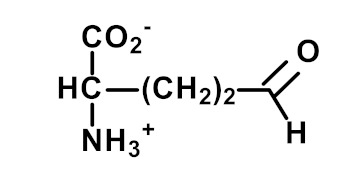 Glutamic semialdehyde (GSA)	Major “protein carbonyl” formed by the oxidative deguanidylation of arginine and oxidative ring-opening of proline [36]	Plasma protein: 0.64 ± 0.33 mmol/mol arg [10]
	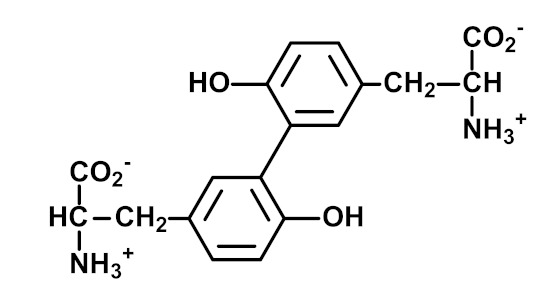 Dityrosine (DT)	Oxidative crosslink formed spontaneously in oxidative stress and enzymatically by DUOX [9,23].	Plasma protein: 0.025 (0.019–0.031) mmol/mol tyr [10]. Increased in autism
	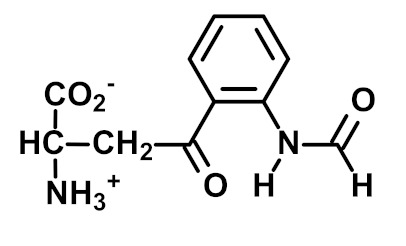 N-Formylkynurenine (NFK)	Formed by the oxidation of tryptophan by hydrogen peroxide, peroxynitrite and hypochlorite [37]. Formed enzymatically by IDO involved in immunoregulation, inflammation and host defense against infectious disease [38].	Plasma protein: 15.6 ± 1.7 mmol/mol trp [10].
Nitration	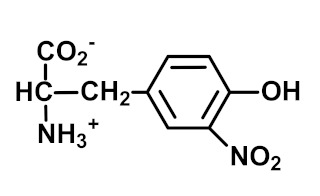 3-Nitrotyrosine (3-NT)	Protein nitration marker. Major proteolysis product of proteins endogenously nitrated by peroxynitrite and nitryl chloride [23,39]. May reflect oxidative stress and/or nitric oxide availability	Plasma protein: 0.0006 ± 0.0004 mmol/mol tyr; increased in diabetes [40].
Citrullination	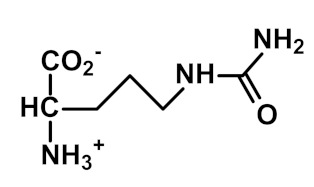 Citrulline residue	Citrullinated protein (CP). Formed enzymatically from arginine residues by PADs [41]	Plasma CP: 0.053 (0.043–0.091) mmol/mol arg; increased in early-stage arthritis [11]
Transglutamination	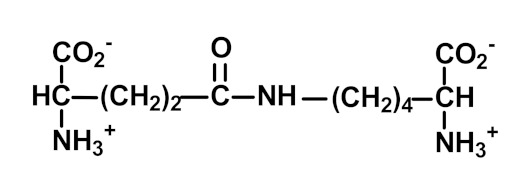 N_ε_(γ-Glutamyl)lysine (GEEK)	Major protein crosslink formed enzymatically by transglutaminases from glutamine and lysine residues [42]	Urinary excretion: 0.42 (0.20–0.93) nmol/mg creatinine [16]

^1^ Data are mean ± standard deviation or median (lower–upper quartile). ^2^ Abbreviated coverage of glycation adducts has been presented previously in [5]. Adapted with permission from Ref. [5]. Copyright year 2021, Elsevier.

**Table 2:** Diagnostic algorithms developed with the AGEomics technique.

Disorder or Disease (Algorithm Development Method)	Analytes (Adduct)	Diagnostic Indication ^1^	Reference
Early-stage arthritis (GLMNET)	Plasma CP, hyp and anti-CCP anti-body status	Diagnostic algorithm for classification of good skeletal health or early-stage arthritis type (OA, RA or non-RA): for Good skeletal health, OA, RA and non-RA, LR+ = 1.6, 5.6, 6.3 and 1.0 and LR− = 0.79, 0.31, 0.47 and 0.99, respectively.	[11]
Early-stage arthritis (Random forests)	Plasma free adducts (FL, CML, CEL, G-H1, MG-H1, 3DG-H, CEL, CMA, GSP, pentosidine; and MetSO, DT, NFK, 3-NT; and hyp and anti-CCP antibody status	Diagnostic algorithm for early-stage arthritis (any type) vs. good skeletal health: LR+ = 8.3 and LR− = 0.11. Diagnostic algorithm for classification of early-stage arthritis type (OA, RA or non-RA): for OA, RA and non-RA, LR+ = 16.1, 7.7 and 5.0 and LR− = 0.06, 0.34 and 0.36, respectively.	[50]
Autism spectrum disorder (Support vector machines)	Glycated plasma protein (CML, CMA, 3DG-H and DT)	Combined in a diagnostic algorithm, gave moderate evidence for presence and borderline moderate/conclusive evidence for absence of ASD; LR+ = 5.7, LR− = 0.095.	[10]
Early-stage decline in metabolic, vascular and renal health (Support vector machines)	Urinary free adduct (FL; and val, age and BMI)	Diagnostic algorithm classifying good health vs. early-stage health decline. LR+, 8.0. 2.8 and 13.2, and LR− 0.24, 0.43 and 0.13 for metabolic, vascular and renal health respectively.	[16]
Diabetic kidney disease risk prediction (X-Gradient boost)	A1C, logACR, FE_CMA_, FEG_-H1_ and [CML]_plasma_	Accuracy 87 ± 4%, sensitivity 74 ± 9%, specificity 91 ± 4%, AUROC 0.90, LR+ 11.0,	[63]

^1^ Interpretation of level of evidence from likelihood ratios: LR+: 1–2, minimal; 2–5, small; 5–10, moderate; >10, large and conclusive. LR−: 0.5–1.0, minimal; 0.2–0.5, small; 0.1–0.2, moderate; <0.1, large and conclusive [62]. Abbreviations: ACR, urinary albumin to creatinine ratio; AUROC, area-under-the-curve of receiver operating characteristic curve; BMI, body mass index; CEL, N_ε_(1-carboxyethyl)lysine; 3DG-H, 3-deoxyglucosone-derived hydroimidazolone structural isomers.
